# Supplementary material for: Disaccharides and Fructooligosaccharides (FOS) Production by Wild Yeasts Isolated from Agave
Source: Foods. 2025 Aug 1;14(15):2714. doi: 10.3390/foods14152714 (PMC12345667; doi:10.3390/foods14152714)
Supplement: Supplementary file 1 [file foods-14-02714-s001.zip › foods-3764085-supplementary.pdf]

## Supplementary Materials

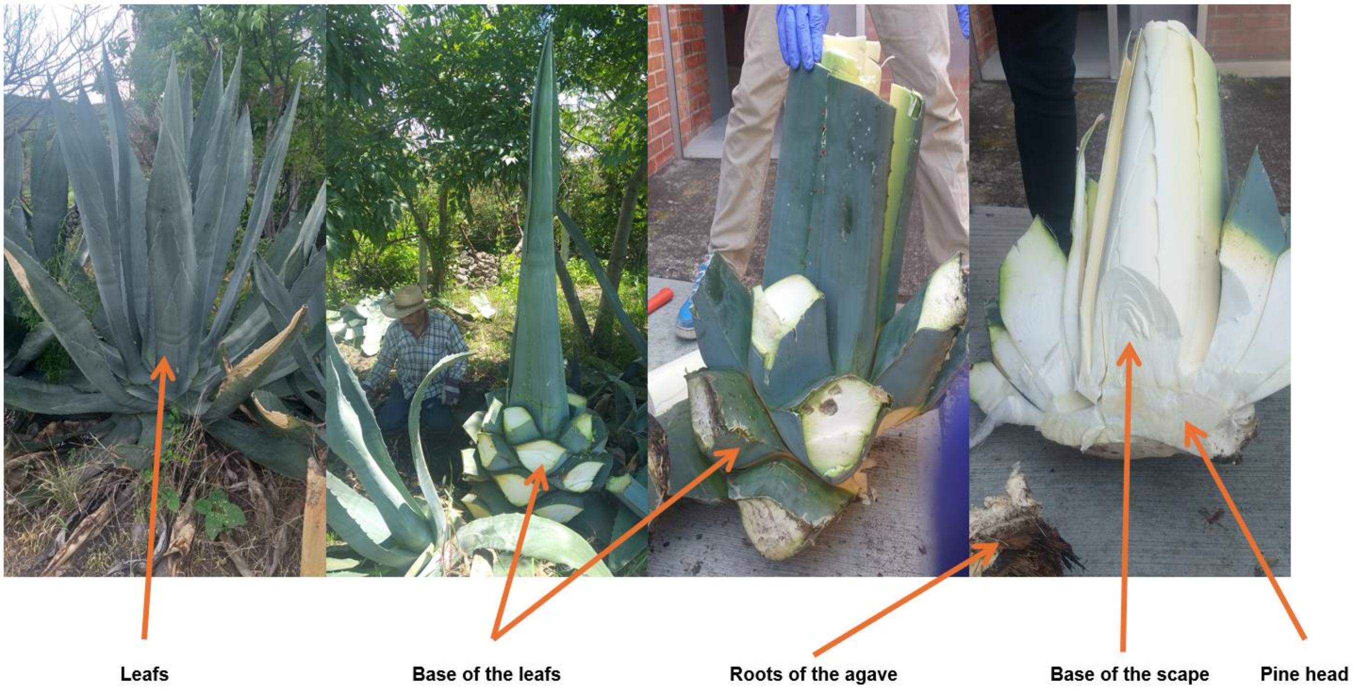


**Figure S1.** The agave was dissected from the leaf (L), base of the leaf (BL), base of the scape (BS), and pine head (P).

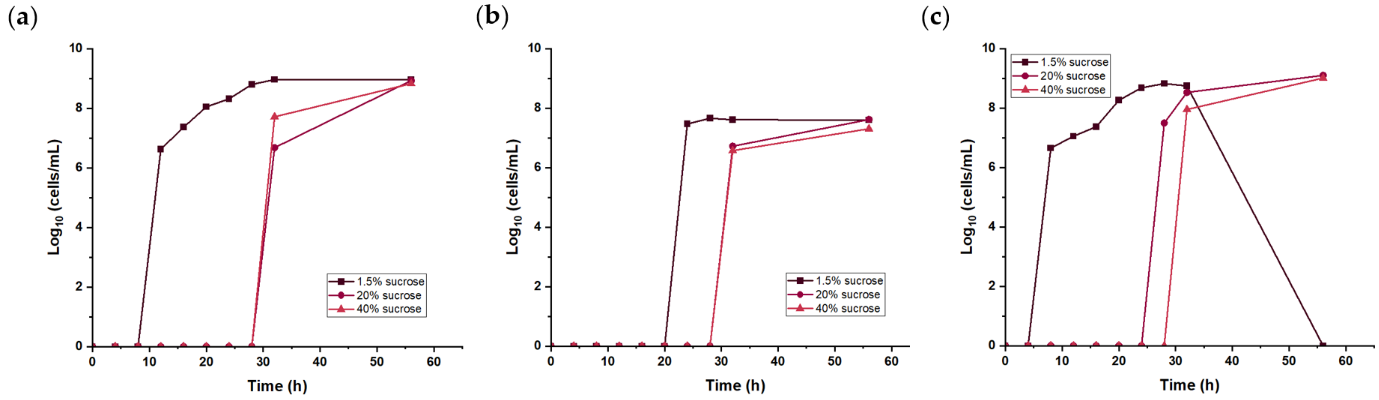


**Figure S2.** Logarithmic plots of the cell growth of (**a**) *P. kudriavzevii* ITMLB97, (**b**) *K. marxianus* ITMLB106, and (**c**) *C. lusitaniae* ITMLB85 with different sucrose concentrations (1.5, 20, and 40%) at 30 °C.

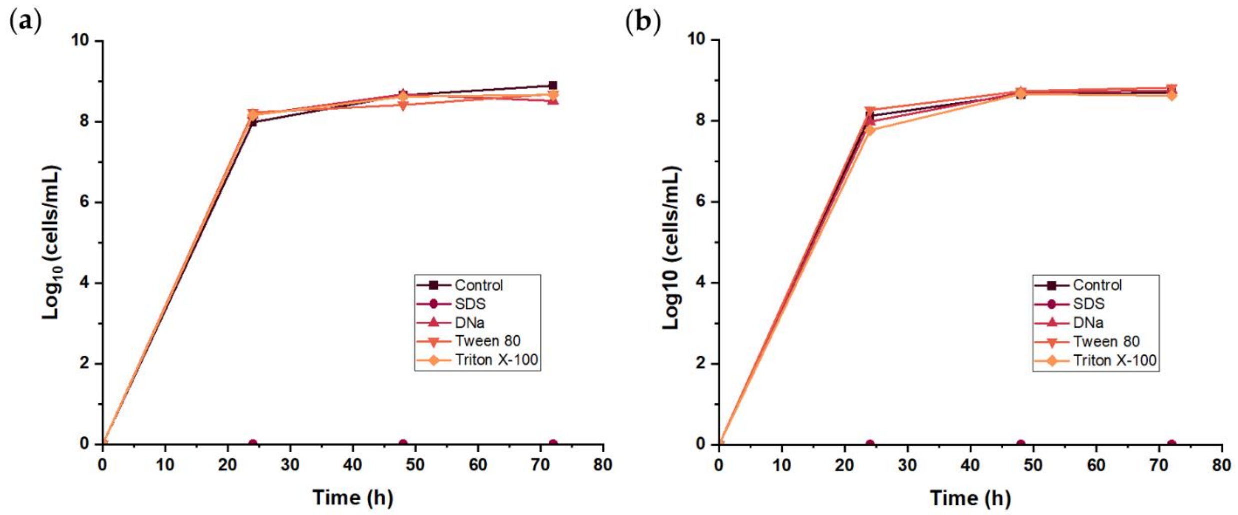


**Figure S3.** Logarithmic plots of the cell growth of (**a**) *P. kudriavzevii* ITMLB97 and (**b**) *C. lusitaniae* ITMLB85 with surfactants at a 20% sucrose concentration in the media over time.

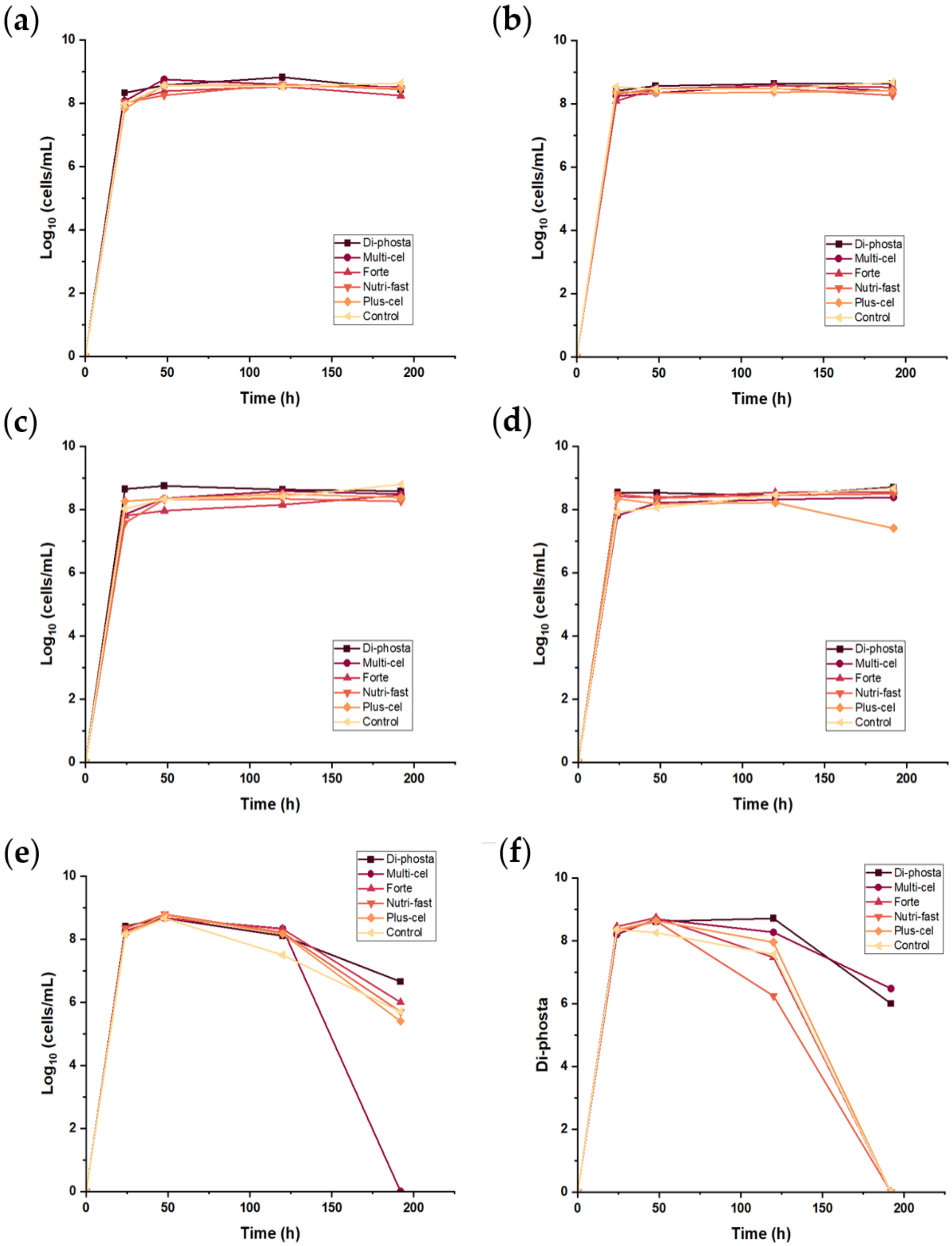


**Figure S4.** Logarithmic plots of the cell growth of (**a**, **c, e**) *P. kudriavzevii* ITMLB97 and (**b, d, f**) *C. lusitaniae* ITMLB85 with different carbon sources and nutrients. *P. kudriavzevii* ITMLB97 used as a carbon source: (**a**) FOS, (**c**) inulin, and (**e**) BS-juice; *C. lusitaniae* ITMLB85 used as a carbon source: (**b**) FOS, (**d**) inulin, and (**f**) BS-juice.

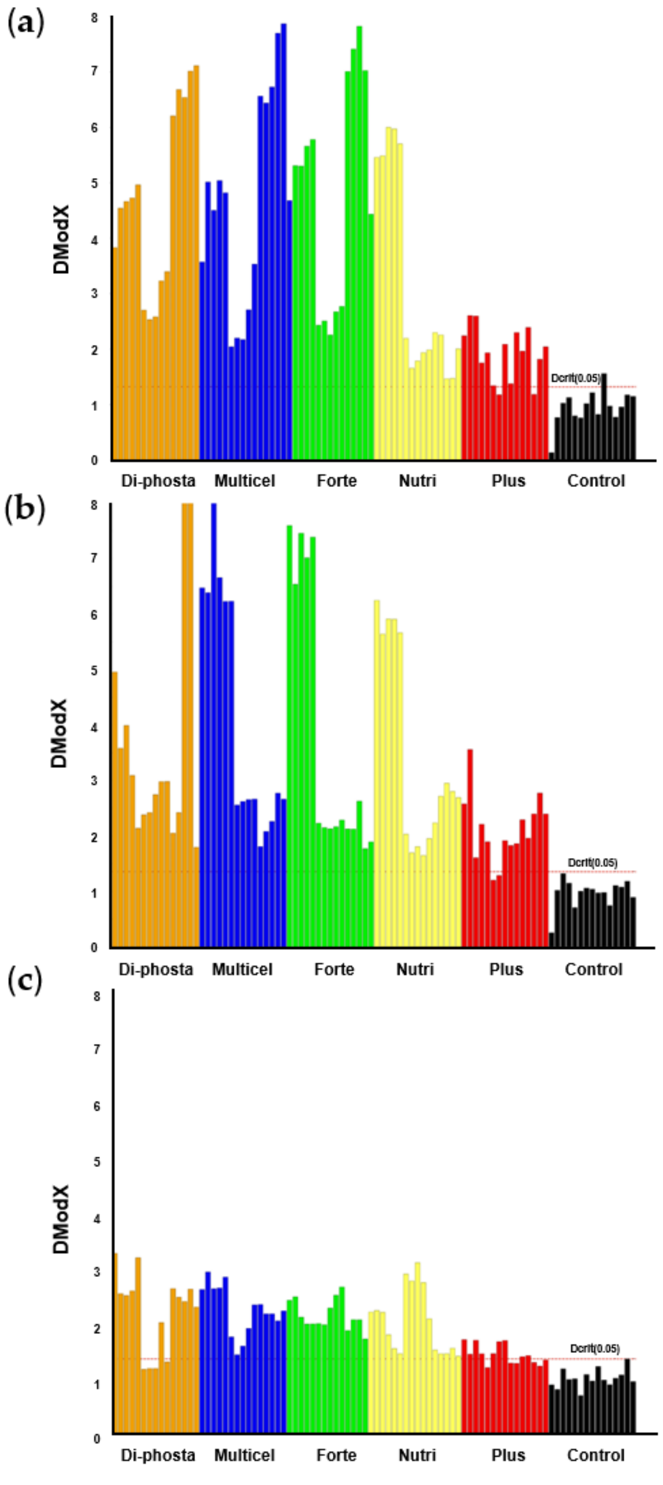


**Figure S5.** Soft independent modelling of class analogy (SIMCA)-analysis of mid infrared spectra of *P. kudriavzevii* ITMLB97 grown with: (**a**) inulin, (**b**) FOS, (**c**), BS-juice as a carbon source in combination with different nutrient formulas (Di-phosta, Multicel, Forte, Nutri-fast, and Plus-cel). The control samples were grown only with the corresponding carbon source without any of the nutrient formulations. Any bar above the Dcrit line is considered as differentiated from the control samples origin model.

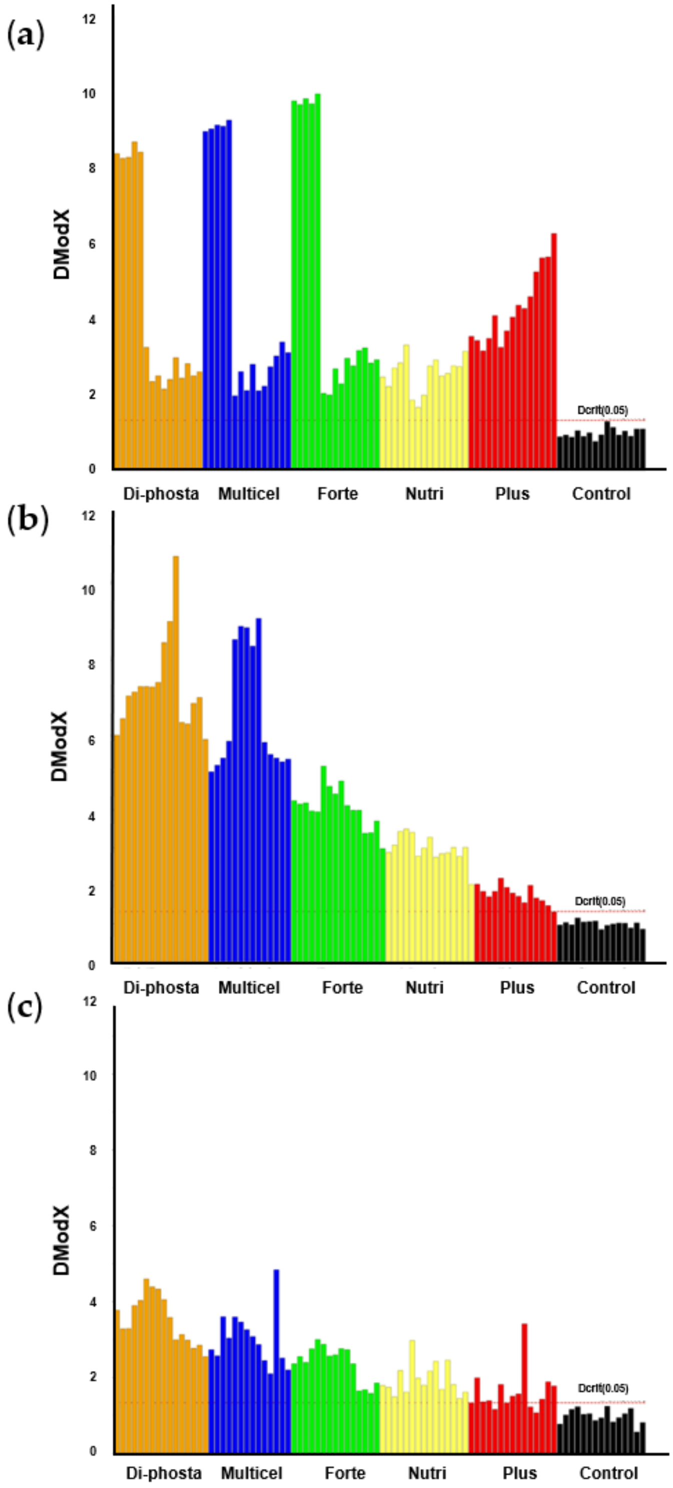


**Figure S6.** Soft independent modelling of class analogy (SIMCA)-analysis of mid infrared spectra of *C. lusitaniae* ITMLB85 grown with: (**a**) inulin, (**b**) FOS (**c**), BS-juice as a carbon source in combination with different nutrient formulas (Di-phosta, Multicel, Forte, Nutri-fast, and Plus-cel). The control samples were grown only with the corresponding carbon source without any of the nutrient formulations. Any bar above the Dcrit line is considered as differentiated from the control samples origin model.
